# Differential expression of cyclooxygenase-2 in metastatic melanoma affects progression free survival

**DOI:** 10.18632/oncotarget.10976

**Published:** 2016-08-01

**Authors:** Elisabetta Panza, Paola De Cicco, Giuseppe Ercolano, Chiara Armogida, Giosuè Scognamiglio, Anna Maria Anniciello, Gerardo Botti, Giuseppe Cirino, Angela Ianaro

**Affiliations:** ^1^ Department of Pharmacy, University of Naples Federico II, Naples Italy; ^2^ Department of Experimental Oncology, National Cancer Institute, G. Pascale, Naples, Italy

**Keywords:** malignant melanoma, cyclooxygenase-2, COX-2^−/−^ mice, progression free survival, metastasis

## Abstract

The possible correlation between cyclooxygenase-2 (COX-2) expression and disease progression in melanoma is still a matter of debate. Analysis of COX-2 expression in 45 lymph node melanoma metastases demonstrates a significant correlation between the percent of expression and progression free survival (PFS). A positive COX-2 expression ≥10% (COX-2^high^), as opposite to a positive expression ≤9% (COX-2^low^), translated into a striking significant reduction of PFS of about 3 years. The reduction in PFS correlated neither with BRAF^V600E^ nor with NRAS^Q61^ expression in the analyzed samples. This concept was reinforced by the finding that tumour development in COX-2^−/−^ mice was almost blunted. Similarly, inhibition of COX-2 protein expression in human melanoma cell lines, by using siRNAs technology as well as selective inhibition of COX-2 activity by celecoxib, reduced cellular proliferation and invasiveness. In conclusion we show that COX-2^high^ is a negative prognostic factor in metastatic melanoma. Our study also clarifies that the uncertainty about the role of COX-2 in metastatic malignant melanoma, found in the current relevant literature, is probably due to the fact that a threshold in COX-2 expression has to be reached in order to impact on cancer malignancy. Our findings suggest that COX-2 expression may become an useful diagnostic tool in defining melanoma malignancy as well as argue for a possible therapeutic use of NSAID as *add on* therapy in selected cases.

## INTRODUCTION

Inflammation has emerged as a major factor promoting cancer development. In the current literature there is an increasing interest for the role played by COX-1 and COX-2, the key rate-limiting enzymes involved in regulation of PGE_2_ synthesis. In particular, the COX-2 isoform has been shown to be constitutively expressed in various cancers, predominantly by stromal cells [1]. In melanoma COX-2 expression has been detected in human specimens and murine models [2, 3]. COX-2 expression has been proposed to be involved in melanoma development and progression [4–6]. More recently this concept has been reinforced by the finding that PGE_2_ –dependent suppression of myeloid cell activation is a potent additional mechanism of tumour immune escape and it is driven by COX-2 derived PGE_2_ [7]. However, at the present stage, there are still conflicting data in the literature concerning the role of COX-2 both in melanoma development and progression. By looking at the different reports we found that studies on malignant melanoma did not address in depth the impact of a different degree of expression of COX-2 in melanoma metastases.

In order to address this issue we performed a retrospective studies on 45 samples from melanoma patients with lymph node metastases. Samples were analyzed for their COX-2 expression in order to define if a threshold expression may represent a negative prognostic factor. A cellular study coupled to an *in vivo* study on COX-2^−/−^ mice has been also performed. Our analysis of COX-2 expression defines a correlation between a threshold expression of COX-2 and a reduction in PFS. Furthermore, we demonstrated that COX-2^−/−^ mice are protected from melanoma development confirming a role for COX-2 also in tumour development. This latter finding has been also established by pharmacological modulation through selective inhibition of COX-2 activity and by a molecular study performed by silencing COX-2 in human melanoma cell line.

## RESULTS

### Human melanoma samples

**Figure 1:**
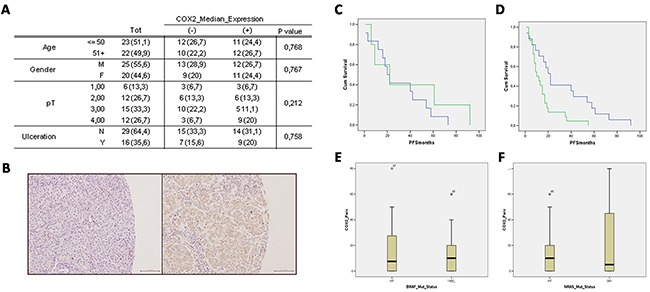
A. Clinical-pathological characteristics of melanoma patients and tumours and relation to COX-2 expression Frequencies are indicated in absolute values (percentage). COX-2 median expression values, where indicated with (−), include negative samples and samples with low COX-2 expression (≤9%); where indicated with (+) include samples with high COX-2 expression (≥10%). Abbreviations: pT (primary tumour), M (male), F (female), Y (present), N (not present). **B-D.** Immunohistochemistry staining of COX-2 and Kaplan–Meier progression free survival curve stratified by median of COX-2 expression. **B.** Immunohistochemistry staining of COX-2: left panel COX-2 negative expression; right panel COX-2 positive expression. Images are 20x. **C.** Kaplan–Meier progression free survival (PFS) curve of cases under the median of COX-2 expression stratified by not expressed (0%; *n*=12; blue line) and low COX-2 expression (≤9%; *n*=5 green line) shows a not significant trend in PFS reduction. **D.** Kaplan–Meier PFS curve of cases stratified by low COX-2 expression (≤9%; *n*= 17; blue line), and high COX-2 expression (≥10%; *n*=22; green line). Kaplan–Meier curve illustrate a significant correlation between COX-2 high expression and PFS. **E-F.** COX-2 expression and BRAFV600E and NRASQ61 mutational status. **(E)** Absence of correlation between COX-2 expression and BRAFV600E mutational status. **(F)** Absence of correlation between COX-2 expression and NRASQ61 mutational status. Data are expressed as BOX-plot distribution.

The histological samples analysed were obtained from 45 lymph node melanoma metastases from the Biobank of the National Cancer Institute G. Pascale. The median age of patients was 50 years, female patients represented 44% (20/45). Primary tumour (pT) grade was distributed as follows: grade 1, 13.3% (6/45); grade 2, 26.7% (12/45); grade 3, 33.3% (15/45); grade 4, 26.7% (12/45). Ulceration was present in 35.6% (16/45) of the samples analyzed (Figure 1A).

### Higher COX-2 expression percentage in lymph node metastases correlates with negative progression free survival (PFS) outcome

COX-2 expression was evaluated in all 45 samples. COX-2 immunoreactivity was detected in 23 out of 45 (51%) lymph node metastases samples. A representative image of COX-2 negative staining is reported in Figure 1 (panel B, left) *vs* the positive staining (panel B, right).

In order to verify if the percent of expression of COX-2 does play a role in melanoma malignancy evaluated as PFS, we compared negative samples *vs* samples with low COX-2 expression (COX-2^low^) set as cut off at up to ≤9%. As it can be seen in Figure 1 panel C, plotting samples with null COX-2 expression (blue line) vs COX-2^low^ expression (≤9%; green line) there was a not significant trend in PFS reduction. Next, positive samples were separated into two new sub-groups, one where COX-2 expression was ≥10%, defined as COX-2^high^ expression (green line), and a second where COX-2 expression was ≤9%, defined as COX-2^low^ (blue line). When we plotted the data we found that the COX-2^high^ expression group showed a striking negative correlation with PFS. Indeed, patient with COX-2^high^ had a reduction in PFS of 35 months (almost 3 years).

### BRAF and NRAS mutational status does not correlate with COX-2 expression in lymph node metastases

In order to verify if melanoma most frequent mutations could influence the data outcome we characterized the NRAS^Q61^ and BRAF^V600E^ mutations. NRAS^Q61^ was present in 8.9% of patients (n = 4/45), while activating BRAF^V600E^ mutations, as expected, was more represented and found in 64.4% of patients (n = 29/45). Interestingly COX-2 expression did not correlate with BRAF^V600E^ (p=0,768) or with NRAS^Q61^ (p=0,934) mutational status (Figure 1E-1F).

### B16-F10 murine cell induced melanoma is blunted in COX-2^−/−^ mice

**Figure 2:**
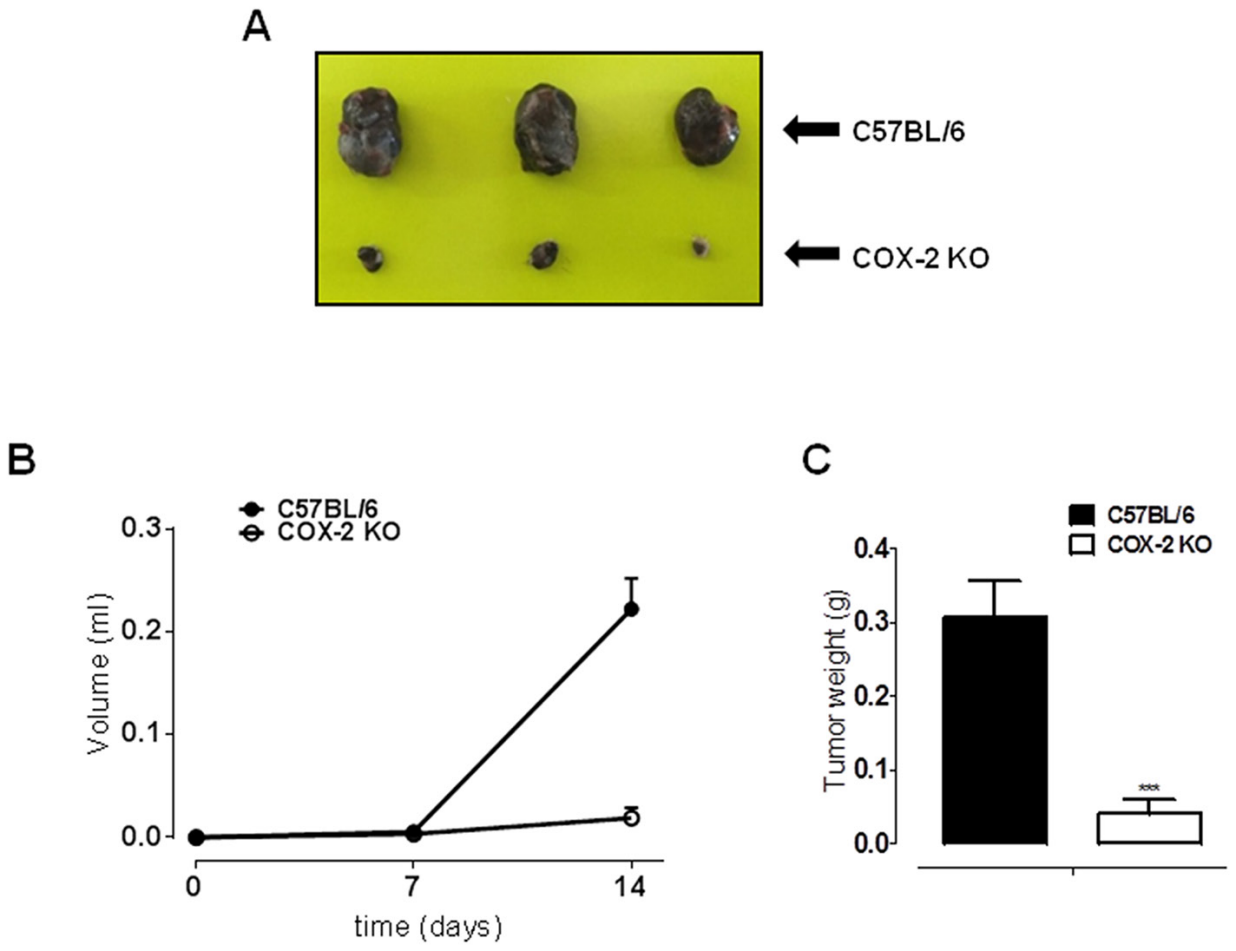
COX-2 is essential for melanoma development in mice B16-F10 murine cells were subcutaneously injected into the right flank of COX-2^−/−^ mice (n=10). C57Bl/6J mice were used as littermate control (n=10). **A.** representative image of tumour sizes. **B.** tumour development expressed as tumour volume. A marked and significant reduction in tumour volume by 91% was observed in COX-2^−/−^ mice (○) (P < 0.001) as compared to littermate mice (•). **C.** tumour weight is significant reduced by 87% (P<0.001) in COX-2^−/−^ mice (open square) as compared to littermate control (filled square).

To investigate the role of COX-2 also in melanoma development rather than in metastasis progression, we performed a reverse translational approach using COX-2^−/−^ mice. Toward this aim, we used the most widely acknowledged experimental model to study melanoma development *in vivo* [8]. The tumour was implanted by subcutaneous injection of B16-F10 murine cells in the right flank of COX-2^−/−^ mice and littermate controls C57Bl/6J. Tumour development in COX-2^−/−^ mice was reduced in volume by 91% and in wet weight by 87% (P<0,001; n=10) (Figure 2A-2C).

### Expression of COX-1 and COX-2 in human melanoma cell lines

**Figure 3:**
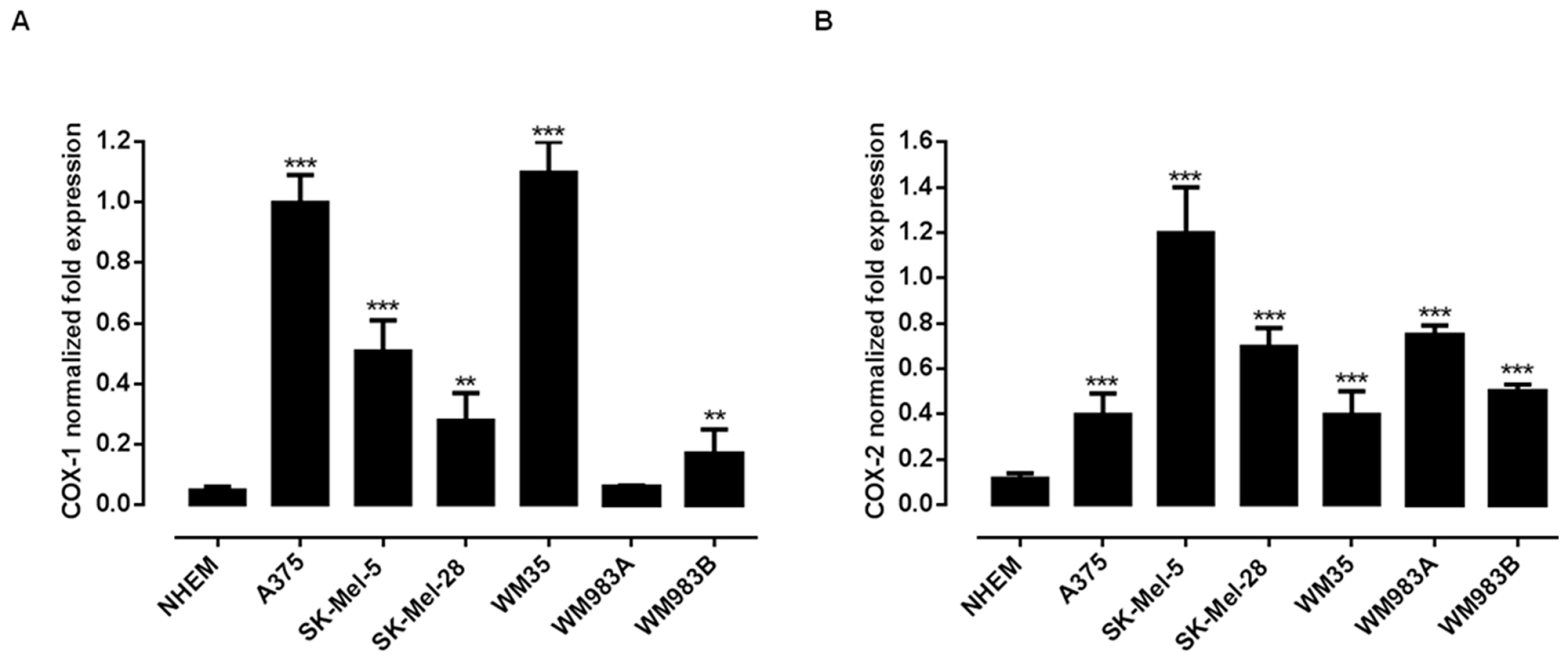
Expression of COX-1 and COX-2 in human melanoma cell lines A quantitative real-time PCR (qPCR) analysis to evaluate the expression levels of both COX-1 **A.** and COX-2 **B.** genes was performed on normal human epidermal melanocytes (NHEM) and on the melanoma cell lines A375, Sk-Mel-5, Sk-Mel-28, WM35, WM983A and WM983B. All human melanoma cell lines expressed both enzymes but with different level of expression. **P<0.01; ***P<0.001 vs NHEM. The housekeeping gene (ribosomal protein S16) was used as an internal control to normalize the ct values.

In order to gain further insights into the role of COXs in human melanoma we decided to operate a pharmacological modulation study by using different melanoma cell lines namely A375, SK-MEL-5, SK-MEL-28, WM35, WM983A, WM983B. The expression levels of both COX-1 and COX-2 genes in normal human epidermal melanocytes (NHEM) and in the cell lines selected were evaluated by performing a quantitative real-time PCR analysis. All three cell lines showed an increased expression of COX-1 and COX-2 as compared to NHEM. Indeed, COX-1 or COX-2 expression was always as minimum triplicated in all cell lines examined. What is of particular interest is the finding that the expression level of both COX-1 and COX-2 appears to reciprocal compensate within the melanoma cell line (Figure 3A, 3B). Indeed, by looking at the Figure 3A and 3B it appears that within each single cell line analyzed the ratio between the two isoforms is always about 1:2. The highest level of COX-2 expression was exhibited by SK-Mel-5 and thus this cell line was selected for the silencing experiments.

### Selective inhibition of COX-2 activity and expression reduces human melanoma cell proliferation and invasiveness

**Figure 4:**
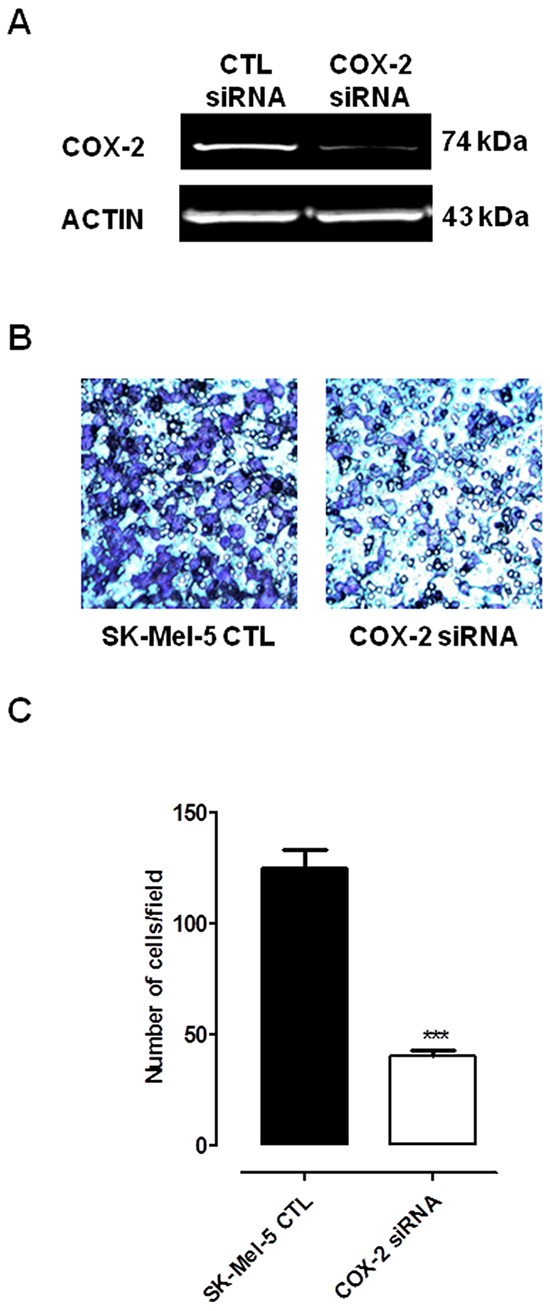
Silencing of COX-2 gene in Sk-Mel-5 cells significantly decreases melanoma cell invasiveness Sk-Mel-5 cell were transfected with COX-2 siRNA to knockdown COX-2 expression. **A.** transfection of Sk-Mel-5 cells by COX-2 siRNA resulted in marked reduction in the levels of COX-2 protein in cells as confirmed by western blot analysis. A significant reduction of cell invasiveness **B, C.** versus control siRNA-treated cells was observed. Each column is the mean ± SEM of three independent experiments, each performed in quadruplicate P<0.001 vs CTL.

To investigate on the effect of COX-2 inhibition on melanoma cell proliferation we choose celecoxib, a selective COX-2 inhibitor, and compared the effect versus naproxen, a non selective COX inhibitor. Usually, concentrations of celecoxib required to induce apoptosis of cultured cells range from 25–100 μmol/L, thus we selected these concentrations to run our proliferation assays. As shown in Table 1 celecoxib, but not naproxen, inhibited the growth of all cell lines tested in a time and concentration-dependent manner. Celecoxib and naproxen did not inhibit proliferation of NHEM (data not shown). To further address the role of COX-2 versus COX-1 we transfected SK-Mel-5 cells with siRNA for COX-2. The knockdown of COX-2 expression in cells after silencing was confirmed by western blot analysis (Figure 4A). As expected, COX-2 silencing significantly reduced cell invasiveness as compared to control (Figure 4B-4C).

**Table 1:** Effect of celecoxib and naproxen on A375, Sk-Mel-5 and Sk-Mel-28 melanoma cells proliferation

(A) 24h			
Cell line	CTL	celecoxib 100μM	naproxen 100μM
Sk-Mel28	0.295±0.002	0.155±0.005***	0.293±0.01
Sk-Mel5	0.317±0.009	0.186±0.01**	0.321±0.01
A375	0.331±0.01	0.206±0.009*	0.342±0.02
**(B)** 48h			
Sk-Mel28	0.503±0.01	0.182±0.01***	0.509±0.01
Sk-Mel5	0.504±0.03	0.421±0.01**	0.691±0.04
A375	0.569±0.01	0.458±0.005	0.621±0.02
**(C) 72h**			
Sk-Mel28	0.798±0.02	0.235±0.01***	0.781±0.005
Sk-Mel5	0.660±0.02	0.413±0.02**	0.686±0.08
A375	0.779±0.03	0.478±0.001**	0.714±0.03

Growth inhibition was measured using the MTT assay and is expressed as OD values at 24-48-72h. Celecoxib, but not naproxen, inhibited the growth of all melanoma cells at all times considered. Control (CTL). Experiments were run in triplicate, each performed in quadruplicate (**P<0.01; ***P<0.001 vs CTL).

## DISCUSSION

Our analysis of COX-2 expression, carried out on 45 lymph node metastases from different primary melanoma sites, indicates that the COX-2 expression level influences human melanoma malignancy. However, if data are analyzed to search a close correlation between COX-2 expression and PFS, the results obtained do not give a clear outcome. Indeed, if the samples analyzed are simply divided into COX-2 negative- *vs* COX-2 positive the correlation with PFS is present but it is not remarkable. By performing a series of differential analysis on the percent of expression of COX-2 we found that when samples analysed were separated into two sub-groups, namely COX-2^high^ where COX-2 expression was ≥10%, and COX-2^low^ where COX-2 expression was ≤9%, a striking difference was evident. Actually, the COX-2^high^ expression group showed a significant negative correlation with PFS which was reduced by 35 months (almost 3 years) in COX-2^high^ patient. Therefore, the level of COX-2 expression, determined as COX-2^high^, represents a trade off versus a COX-2 negative significant contribute to melanoma malignancy. It is possible to hypothesize that COX-2^high^ tumour promoting effect may be driven by COX-2 derived PGE_2_ as a potent tumour immune escape mechanism. It is well known, and exhaustively explored in the current literature, that COX-2 has a fundamental function in driving tumorigenesis through the production of prostaglandins, which in turn act directly on cancer cells to inhibit apoptosis and enhance cell migration. In fact, prominent among tumor-sustaining mediators is PGE_2_, a prostanoid lipid associated with enhancement of cancer cell survival, growth, migration, invasion, angiogenesis, and immunosuppression [1]. Recently, Zelenay et al., by using an arbitrary cut off of microarray expression data in melanoma biopsies, demonstrate a positive correlation among COX-2 and the levels of some specific tumour promoting factor [7]. The same mechanism [7] may apply to our COX-2^high^ samples. In fact, we can speculate that, the overcoming of a specific threshold in COX-2 expression, allows the tumour to become more ‘aggressive’ with an higher probability of malignant metastasis.

In order to verify if melanoma most frequent mutations could influence the data outcome we characterized the NRAS^Q61^ and BRAF^V600E^ mutations in the samples analyzed. COX-2 expression neither correlated with BRAF^V600E^ nor with NRAS^Q61^ mutational status. Therefore, since COX-2^high^/PFS correlation is affected by neither NRAS^Q61^ nor BRAF^V600E^ mutations it appears that COX-2^high^ is a variant non affected by these mutations, at least in our study.

In order to investigate on the role of COX-2 also in melanoma development rather than in metastasis progression, we performed a reverse translational approach using COX-2^−/−^ mice. The results obtained were remarkable since the lack of COX-2 almost blunted tumour development as opposite to background mice. This finding, - attained with mice with a competent immune system-, supports a key role for COX-2 *vs* COX-1 in melanoma development. However, since the removal of COX-2 gene does not completely abolish tumour development, COX-1 residual contribute cannot be ruled out. In order to gain further insights into the role of COXs in human melanoma we decided to operate a pharmacological modulation by using different human melanoma cell lines. All cell lines showed an increased expression of COX-1 and COX-2 as compared to NHEM. The level of expression of both enzymes varied among the cell lines studied suggesting that expression of COX-1 *vs* COX-2 appears to reciprocal compensate within melanoma cells. In other words, when COX-1 expression is lower COX-2 is doubled and *viceversa*. However, in order to further define the major role of COX-2 *vs* COX-1 we performed a pharmacological modulation study in vitro. We choose as selective COX-2 inhibitor celecoxib and compared its effect to naproxen, a NSAID less than 5 fold selective for COX-2 [9, 10]. Celecoxib was selected since is known to exhibit the greatest potency among COX inhibitors for growth inhibition [11]. Celecoxib, but not naproxen, inhibited the growth of all cell lines tested in a time and concentration-dependent manner further supporting a major role for COX-2 in melanoma development. We next transfected SK-Mel-5 cells with siRNA for COX-2. As expected COX-2 silencing significantly reduced both cell proliferation and invasiveness as compared to control siRNA. Thus, in melanoma cell lines, COX-2 increased expression appears to be a “cellular tool” to increase their ability to invade the host.

In conclusion, we have shown that when COX-2 expression rises above a threshold level, such as in the COX-2^high^ samples, it is a negative prognostic factor for human metastatic melanoma. This finding can explain the conflicting results present in the current literature and help to delineate when COX-2 can be defined a negative prognostic factor. However we have to consider that the present study shows some limitations due to the retrospective analysis but it offers the rationale to design a more accurate prospective study.

In addition, we show pre-clinical evidences that COX-2 plays a role also in melanoma development as demonstrated in vivo by using COX-2^−/−^ mice. Finally, our findings suggest that COX-2 expression may become an useful diagnostic tool in defining melanoma malignancy as well as argue for a possible therapeutic use of NSAID as *add on* therapy in selected cases

## MATERIALS AND METHODS

### Patients and specimens

The retrospective study samples consisted of 45 metastatic lymph node samples obtained from melanoma patients who underwent surgical resection from September 2001 to January 2009 in Istituto Nazionale per lo Studio e la Cura dei Tumori “Fondazione G. Pascale”, Naples (Italy) and were enrolled in a specific clinical protocol where all the 45 patients were diagnosed with lymph node metastases after the first surgery (“in progress disease”). PFS was selected as primary outcome. The melanomas were divided according to the AJCC TNM classification for melanoma staging into four groups pT1 (*n* = 6 melanomas), pT2 (*n* = 12 melanomas), pT3 (*n* = 15 melanomas) and pT4 (*n* = 12 melanomas). The number of patients in the different sub-groups were: for levels of COX-2 expression <=9% (*n*=23); for levels of COX-2 expression >10% (*n*=22). Data reported in Figure 1 (panel C and D) are related to 39 patients since PFS data were not available for 6 out of 45 patients.

### Tissue micro-array

Tissue micro-array (TMA) was built using the two representative areas from each single case. All tumours areas were selected by two experienced pathologists (GB, AMA). Finally, two tissue cylinders (diameter 1 mm) were punched from morphologically representative tissue areas of each donor tissue block and brought into one recipient paraffin block using a semi-automated tissue arrayer (Galileo TMA CK3500, Integrated System Engineering srl, Milan, Italy).

### Immunohistochemistry analysis

Immunohistochemical staining was carried out on TMA 4-μm section to evaluate the expression of COX-2 marker. Briefly, paraffin slides were deparaffinized in xylene and then rehydrated through alcohols gradient. Antigen retrieval was performed by decloaking chamber™ (Biocre Medical) in 0.01 M citrate buffer for 10 min. After peroxidase and protein block (BSA 5% in 1X PBS), the slides were incubated with primary antibody to human COX-2 (D5H5 XP® Cell Signaling). Antigen expression was evaluated independently and blindly by two experienced pathologists (GB/AMA) using light microscopy. The percentage of cancer cells with cytoplasmic staining was determined by counting the number of positive cells as a fraction of the total number of cancer cells in tissues cores at ×400 magnification as follow:

$$\%\text{ cancer cells with cytoplasmic COX}-2\text{ staining}=\frac{\text{positive cells}}{\text{total n}{}^{\circ}\text{ cancer cells}.}$$

The median value of positive expression (9%) was used as the cut-off point for statistical analyses to distinguish tumours with negative or low COX-2 expression (≤9%; COX-2^low^) from tumours with high COX-2 expression (≥10%; COX-2^high^).

### Cell culture and reagents

NHEM were purchased from Lonza (Walkersville, MD, USA) and were grown in Melanocyte growth medium 2 (Lonza). The melanoma cells lines B16/F10, Sk-Mel-5 and Sk-Mel-28 were purchased from IRCCS AOU San Martino – IST (Genova, Italy), A375 from Sigma-Aldrich (Milan, Italy) and were cultured in Dulbecco's modified Eagle's medium (DMEM) containing 10% fetal bovine serum, 2 mmol/L L-glutamine, 100 μmol/L non essential amino acids, penicillin (100 U/mL), streptomycin (100 μg/mL) and 1 mmol/L sodium pyruvate (all from Sigma-Aldrich, Milan, Italy). WM35, WM983A and WM983B were from Rockland (Limerick, Ireland) and were cultured in Tumor Specialized Media (1:5 Leibovitz's – MCDB153), containing 2% Inactivated FBS and 1,68 mM CaCl_2_. Cells were grown at 37°C in a humidified incubator under 5% CO_2_. All cell lines used in this study were characterized by the cell bank were they were purchased. Celecoxib (Selleck Chemicals, Munich, Germany) and naproxen (Sigma-Aldrich, USA) were solubilized in H_2_O.

### RNA purification and quantitative real-time PCR (qPCR)

Total RNA was isolated from cells by use of the TRI-Reagent (Sigma-Aldrich, Milan, Italy), according to the manufacturer's instructions, followed by spectrophotometric quantization as previously described [12]. Final preparation of RNA was considered DNA- and protein-free if the ratio between readings at 260/280 nm was ≥1.7. Isolated mRNA was reverse-transcribed by use of iScript Reverse Transcription Supermix for RT-qPCR (Bio-Rad, Milan, Italy). The quantitative real-time PCR was carried out in CFX384 real-time PCR detection system (Bio-Rad, Milan, Italy) with specific primers (hCOX-1 5′-AAGGTGGCATTGACAAACTCC-3′, 5′-CG CCAGTGATCCCTGTTGTT-3′; hCOX2 5′-TAAGTGC GATTGTACCCGGAC-3′, 5′-TTTGTAGCCATAGTCA GCATTGT-3′) by the use of SYBR Green master mix kit (Bio-Rad, Milan, Italy). Samples were amplified simultaneously in triplicate in one-assay run with a non-template control blank for each primer pair to control for contamination or primer-dimers formation, and the ct value for each experimental group was determined. The housekeeping gene (ribosomal protein S16) was used as an internal control to normalize the ct values, using the 2*^-ΔCt^* formula.

### COX-2 small interfering RNA transfection of SK-Mel-5

For the silencing experiments SK-Mel-5 were seeded onto 96-well plates (2 × 10^3^ cell/well) and transfected the next day, according to the manufacturer's instruction, with PTGS2 Trilencer-27 Human siRNA (OriGene, Rockville, MD, USA) (rCrCrArArUrUrGrUrCrArUrArCrGrArCrUrUrGrCrArGrUGA; rGrGrCrUrArArUrArCrUrGrArUrArGrGrArGrArGrArCrUAT; rGrCrArGrCrUrUrCrCrUrGrArUrUrCrArArArUrGrArGrATT).

The final concentration of the siRNA pool was 10 nM. Forty-eight hours after transfection, cell proliferation was evaluated by MTT assay (see Proliferation assays). The knockdown of COX-2 expression in cells after transfection was confirmed using western blot analysis. The Universal scrambled negative control siRNA duplex was used as negative control.

### Preparation of cell lysates and western blot analysis

Melanoma cells were harvested, washed with cold phosphate-buffered saline and lysed with ice-cold lysis buffer supplemented with protease inhibitors, as detailed previously [12]. Equal amounts of proteins were resolved on 10% Tris–Glycine gels and transferred onto a nitrocellulose membrane. After blocking the non-specific binding sites, the membrane was incubated with the primary antibody (COX-2; cod: 12282; batch 2; diluited 1:1000, Cell signaling, MA, USA) at 4°C overnight. The membrane was then incubated with the appropriate peroxidase-conjugated secondary antibody and the immunoreactive bands were visualized using the enhanced chemiluminescence reagents. To verify equal protein loading, the membrane was stripped and reprobed with anti-b actin antibody.

### Proliferation assay

Cell proliferation was measured by the 3-[4,5-dimethyltiazol-2-yl]-2,5-diphenyl tetrazolium bromide (MTT) assay as previously described [12].

Briefly, the human melanoma cells and the NHEM cells were seeded on 96-well plates (2 × 10^3^ cells/well) and treated with celecoxib (10-100 μM) or naproxen (10-100 μM) for 24-48-72 h before adding 25 μL of MTT (Sigma, Milan, Italy) (5 mg/mL in saline). Cells were thus incubated for an additional 3 h at 37°C. After this time interval, cells were lysed, and dark blue crystals were solubilized with a solution containing 50% N, N-dimethyl formamide and 20% sodium dodecylsulfate with an adjusted pH of 4.5. The optical density of each well was measured with a microplate spectrophotometer (TitertekMultiskan MCC/340), equipped with a 620 nm filter.

### Cell invasion assay

The assay was performed using chambers with polycarbonate filters with 8-μm nominal pore size (Millipore, USA) coated on the upper side with Matrigel (Becton Dickinson Labware, USA). The chambers were placed into a 24-well plate. Two groups of melanoma cells (2.5×10^5^/mL) were harvested and placed in the upper chamber in serum-free DMEM: SK-Mel-5 CTL and siRNA COX-2 transfected SK-Mel-5. The bottom chamber contained DMEM with 10% FBS. After the incubation period (16h), the filter was removed, and non-invaded cells on the upper side of the filter were detached with the use of a cotton swab. Filters were fixed with 4% formaldehyde for 15 min, and cells located in the lower filter were stained with 0.1% crystal violet for 20 min and then washed with PBS. The filters were examined microscopically and cellular invasion was determined by counting the number of stained cells on each filter in at least 4–5 randomly selected fields. Resultant data are presented as a mean of invaded cells ± SD/microscopic field of three independent experiments.

### Animals

Animal care was in accordance with Italian and European regulations on the protection of animals used for experimental and other scientific purposes. Mice were observed daily and humanely euthanized by CO_2_ inhalation if a solitary subcutaneous tumour exceeded 1.5 cm in diameter or mice showed signs referable to metastatic cancer. All efforts were made to minimize suffering. Male C57Bl/6J mice (18-20 g) were purchased from Charles River Laboratories, Inc.

Male COX-2^−/−^ mice, kindly supplied by Dr Jane A. Mitchell, back-crossed for >7 generations onto a C57Bl/6J background were used at 10 to 12 weeks of age. Animals were genotyped before use [13]. Mice were housed at the Animal Research Facility of the Department of Pharmacy of the University of Naples Federico II.

### Induction of subcutaneous B16 lesions

Mice were subcutaneously (s.c.) injected in the right flank with B16-F10 cells (1×10^5^/0.1ml). Tumour size was measured using a digital caliper, and tumour volume was calculated using the following equation: tumour volume=π/6(D1xD2xD3) where D1=length; D2=width; D3= height and expressed as cm^3^ [12].

### Statistical analysis

To analyze the correlation between COX-2 expression and BRAF or NRAS mutation the Mann-Whitney non-parametric test was used. Progression free survival (PFS) curve was calculated with the Kaplan-Meier method and analyzed with the log-rank test. P values less than 0.05 were considered to be statistically significant. Data from all in vivo experiments are reported as the mean ± SEM. Data were analyzed using GraphPad Prism software (GraphPad). Significance was determined using a Student's two-tailed *t* test. Results were considered significant at P value less than 0.05 and are labeled with a single asterisk. In addition, P values less than 0.01 and 0.001 are designated with double and triple asterisks, respectively.
